# Supplementary figures and images for: A Flexible Network of Lipid Droplet Associated Proteins Support Embryonic Integrity of C. elegans
Source: Front Cell Dev Biol. 2022 Apr 4;10:856474. doi: 10.3389/fcell.2022.856474 (PMC9015696; doi:10.3389/fcell.2022.856474)

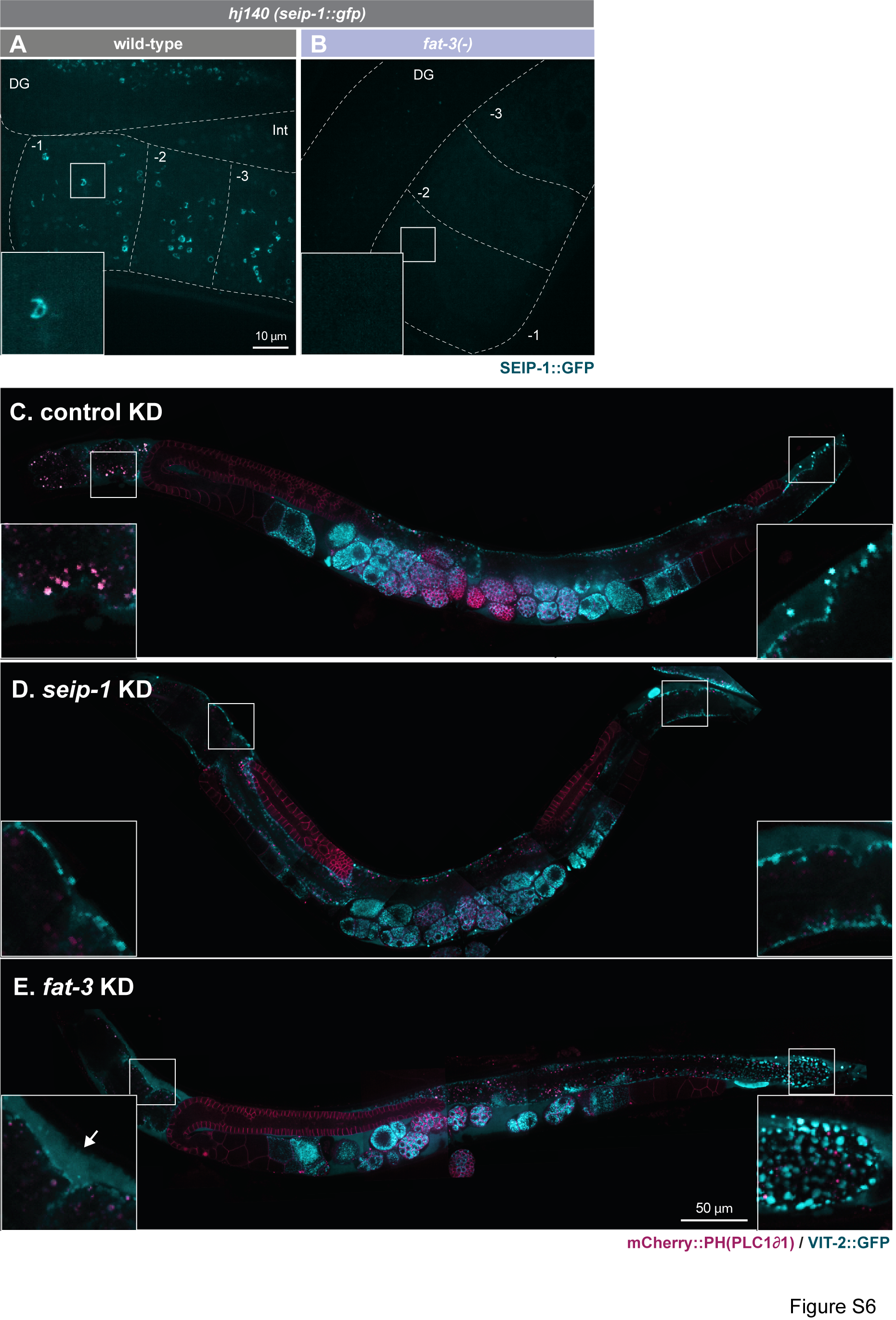

Supplement: Supplementary file 1 [file Image6.TIF]

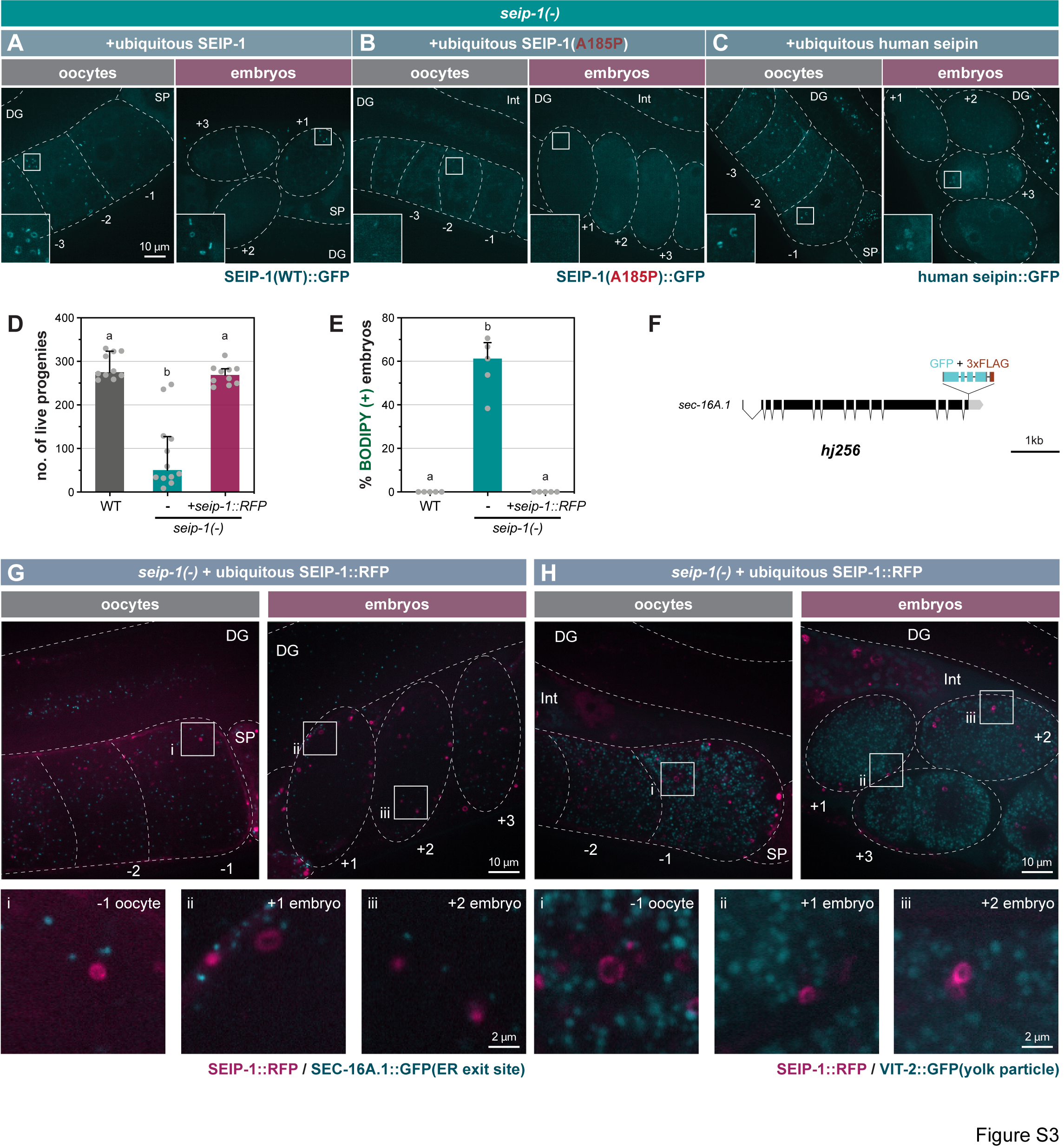

Supplement: Supplementary file 3 [file Image3.TIF]

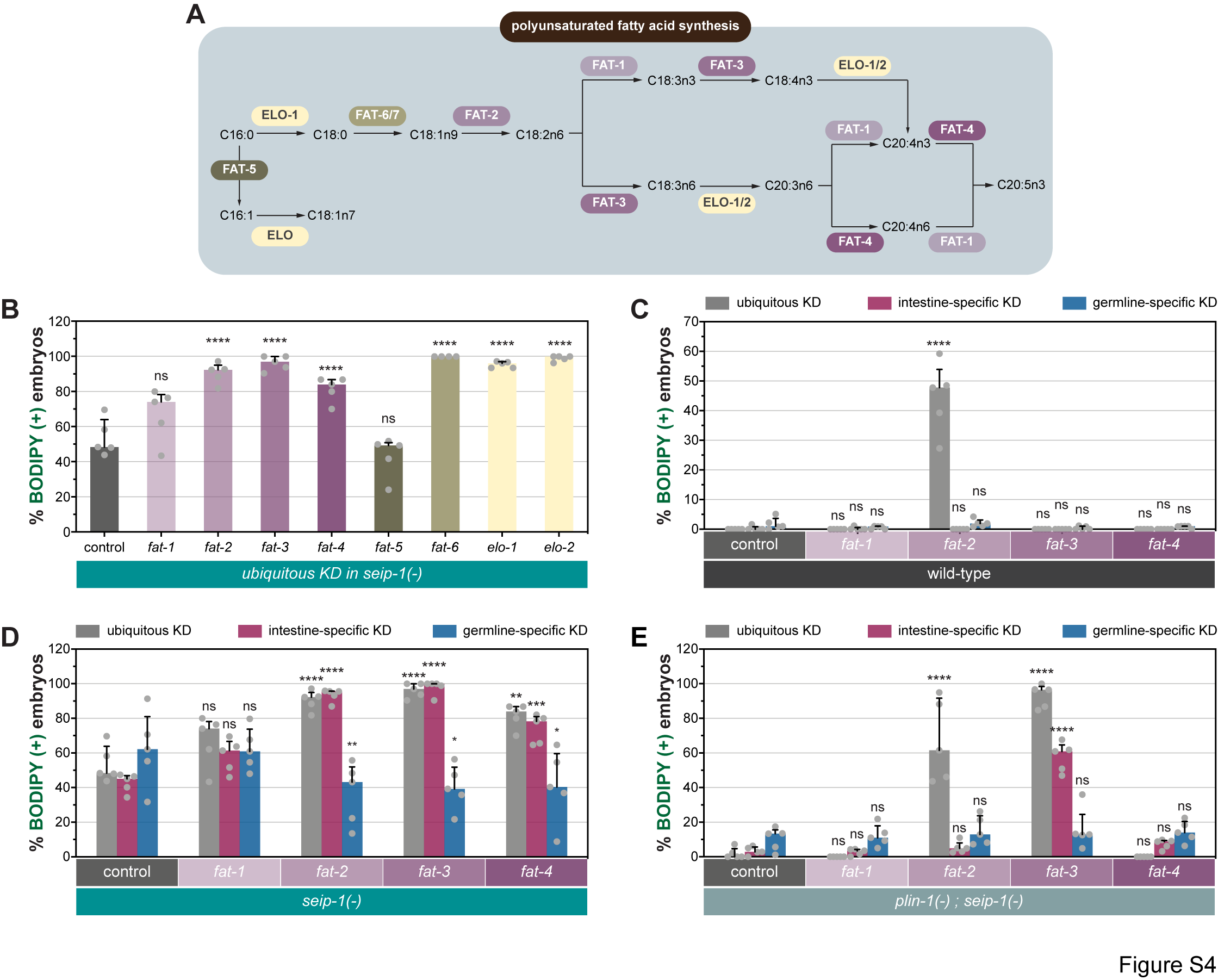

Supplement: Supplementary file 4 [file Image4.TIF]

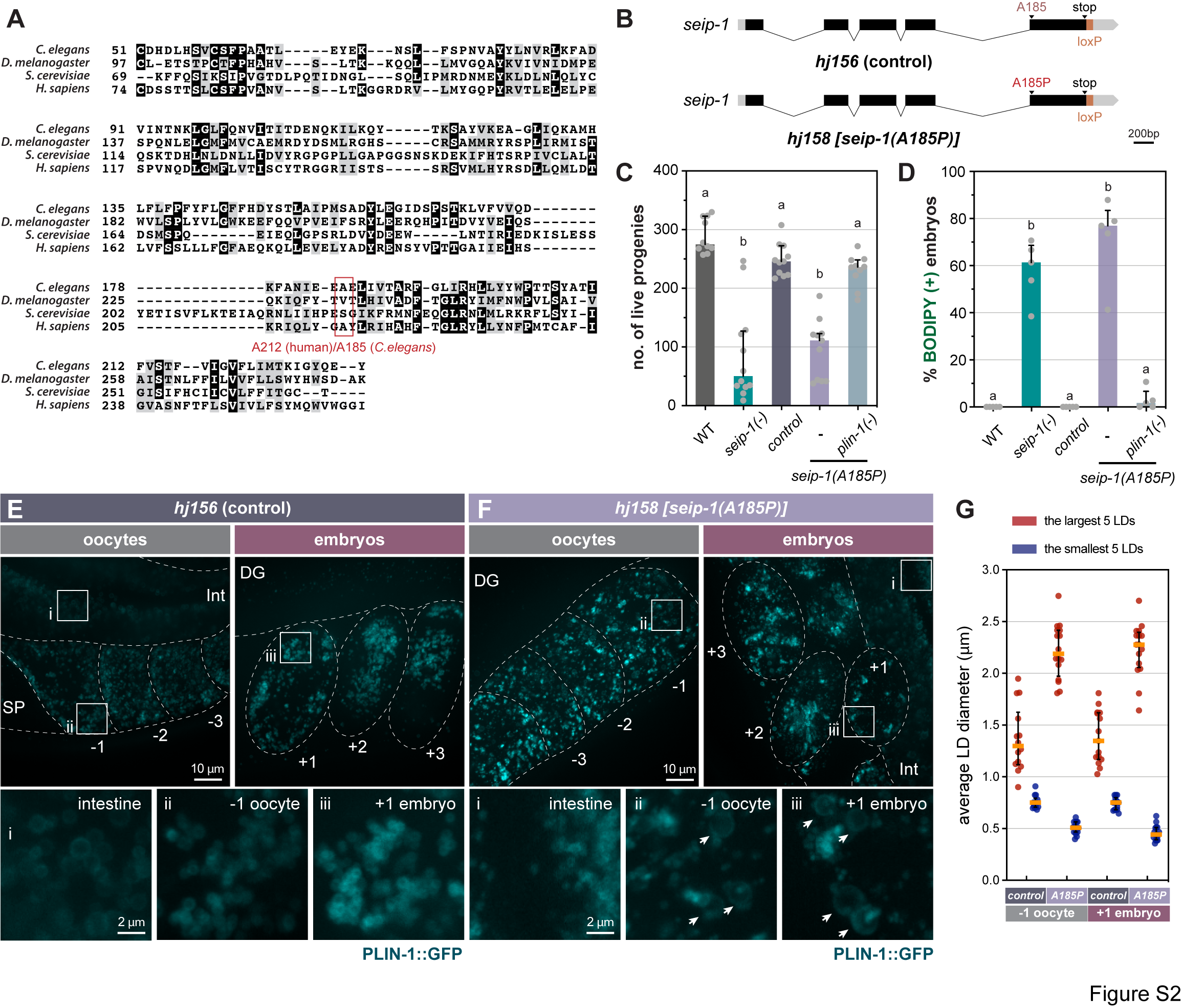

Supplement: Supplementary file 5 [file Image2.TIF]

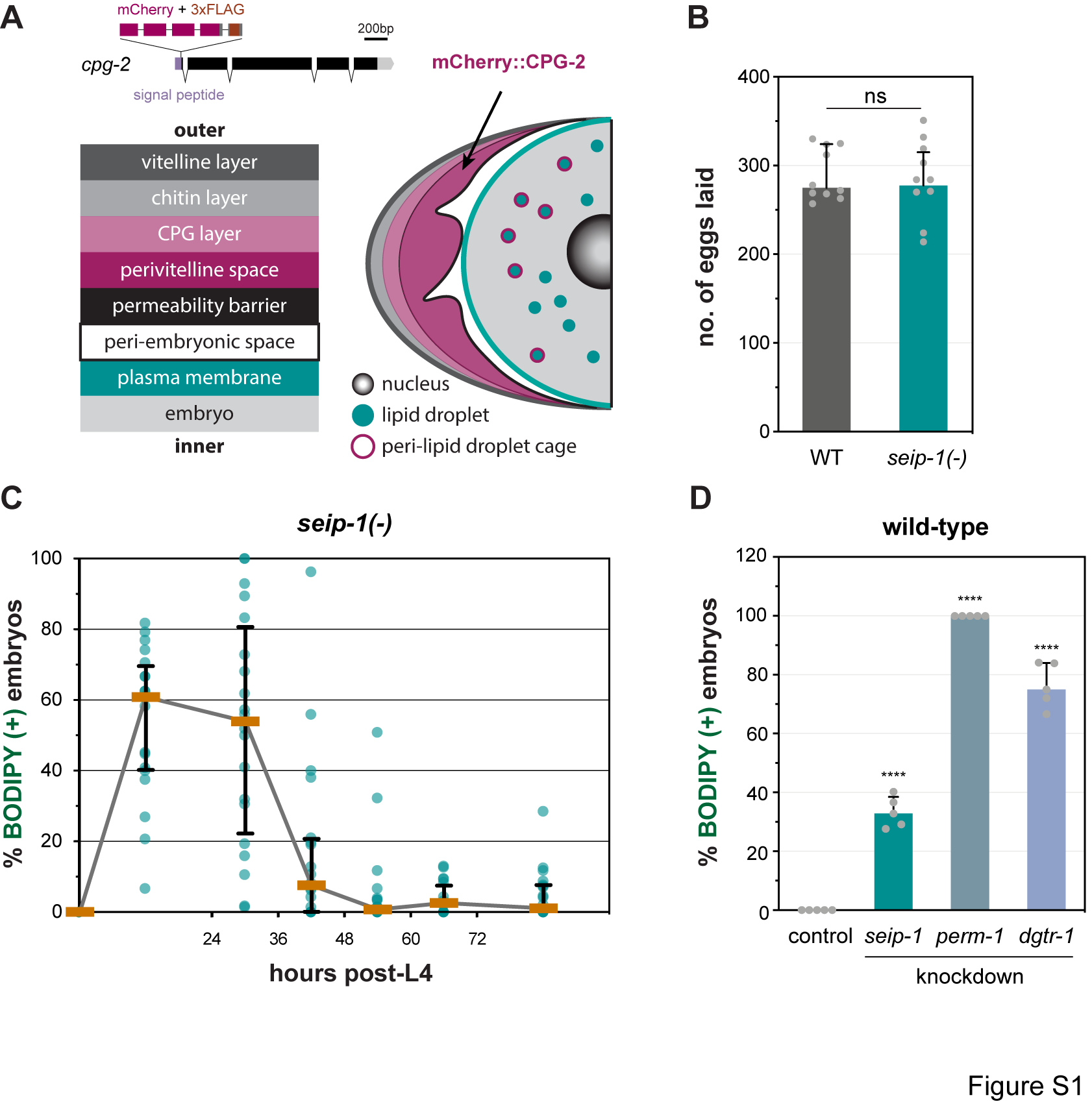

Supplement: Supplementary file 6 [file Image1.TIF]

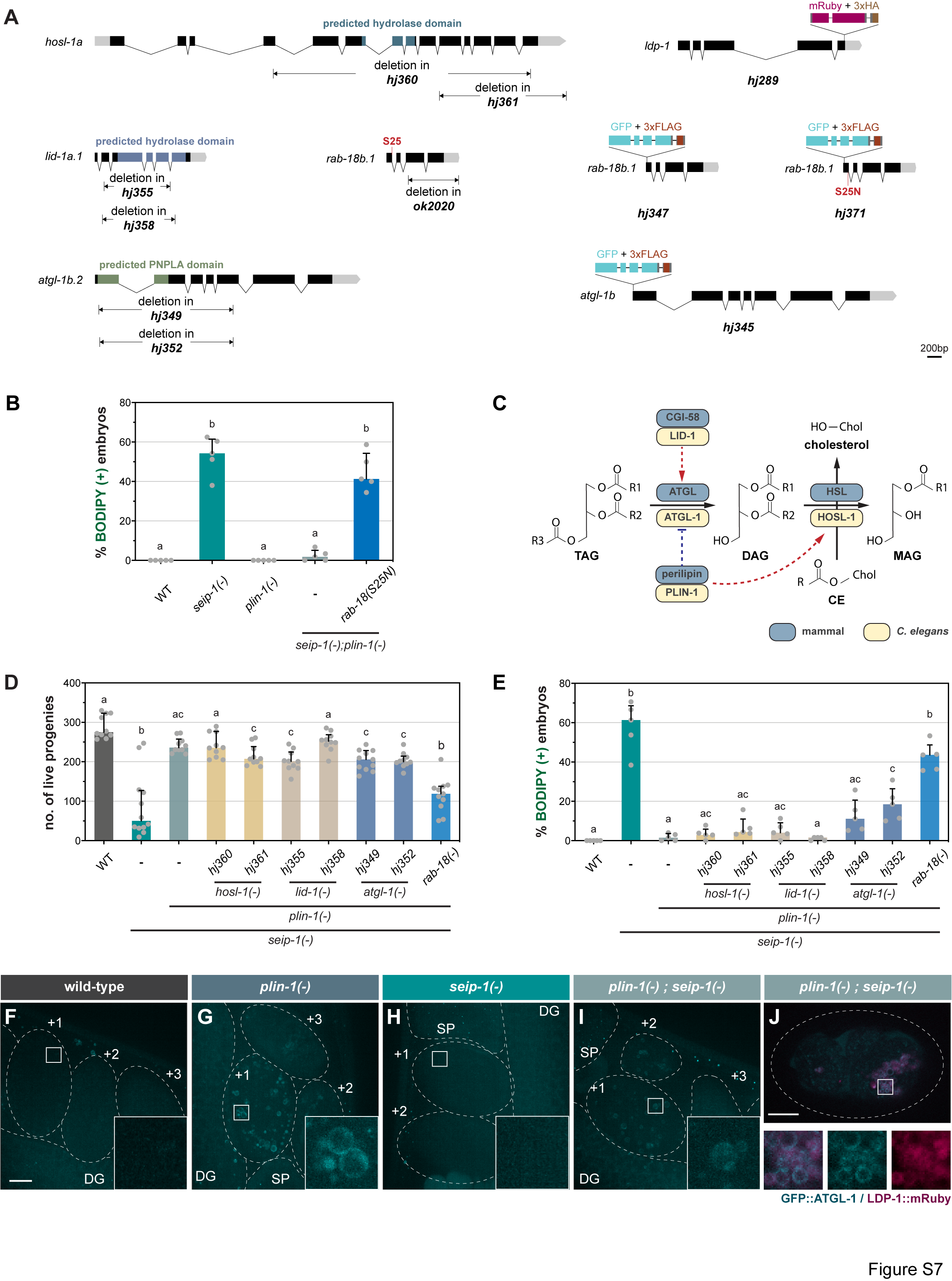

Supplement: Supplementary file 7 [file Image7.TIF]

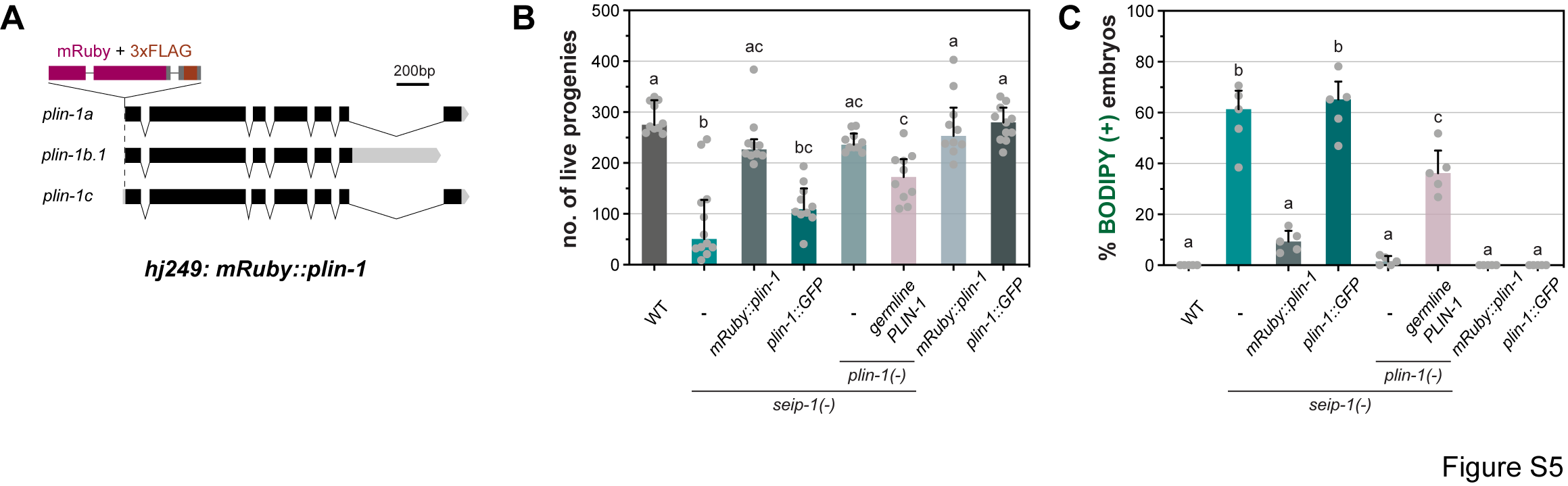

Supplement: Supplementary file 11 [file Image5.TIF]
