# Supplementary material for: A Flexible Network of Lipid Droplet Associated Proteins Support Embryonic Integrity of C. elegans
Source: Front Cell Dev Biol. 2022 Apr 4;10:856474. doi: 10.3389/fcell.2022.856474 (PMC9015696; doi:10.3389/fcell.2022.856474)
Supplement: Supplementary file 8 [file DataSheet1.pdf]

A flexible network of lipid droplet associated proteins support embryonic integrity of *C. elegans*

Supplementary Figure Legends

Figure S1. SEIP-1 supports eggshell integrity of *C. elegans*.

(A) A schematic diagram summarizing the eggshell composition of wild-type *C. elegans*. The design of the *mCherry::3xFLAG::cpg-2* knockin allele is also shown.

(B) Comparison of number of eggs laid by wild type (WT) and *seip-1(-)* animals. At least 10 animals of each genotype were scored. Median with interquartile range is shown (applies to all subsequent bar charts and scatter plots).

(C) The percentage of BODIPY-stained embryos produced by individual *seip-1(-)* adult during the egg-laying period. 20 animals were monitored.

(D) The percentage of BODIPY-stained embryos produced by animals of the indicated genotype, quantified in a defined time window. Five independent biological samples were scored, each with progeny from four 1-day-old adults. For detailed experimental setup, refer to Methods and Materials.

Figure S2. Animals that express SEIP-1(A185P) phenocopy *seip-1(-)* mutants.

(A) Sequence alignment of *C. elegans*, *Drosophila*, *S. cerevisiae*, and human seipin orthologs. The human lipodystrophy-associated A212P mutation in seipin is equivalent to A185P in *C. elegans* SEIP-1.

(B) Schematic representation of specific mutations introduced to the endogenous *seip-1* locus. The two alleles, *hj156* and *hj158*, were engineered by CRISPR/Cas9-mediated HDR with repair templates that only differ at the coding sequence of A185. A loxP scar was left

between the stop codon and 3'-UTR of *seip-1* as a result of removing the self-excising cassette (SEC).

(C) The total number of live progenies from individual animals. At least 10 animals of each genotype were scored. Groups that do not share the same letters are significantly different (ordinary one-way ANOVA with Turkey's multiple comparisons test, $p<0.01$).

(D) As in (C), but with the percentage of BODIPY-stained embryos quantified in a defined time window. Five independent repeats were scored, each with progeny from four 1-day-old adults. Data of the control groups in (C-D) were reproduced from Fig. 1A-B as the measurement was all performed at the same time.

(E) Visualization of LDs using PLIN-1::GFP (*hj178*) in 1-day-old *seip-1(hj156)* (control) adults. mCherry::PH(PLC1δ1) (*itIs44*) labels PM in the germline. mCherry and GFP are pseudocolored magenta and cyan, respectively. Dotted lines mark the boundary between different tissues or embryos. Boxed regions were magnified 5x and displayed at the bottom. A projection of 4.5 μm z stack reconstituted from 10 focal planes is shown. For anatomical positions of the ROI, refer to Figure 2A.

(F) As in (E), but with *seip-1(hj158)* (A185P). Arrows point to aberrantly enlarged LDs.

(G) Average diameter of the largest (crimson) or smallest (navy-blue) five LDs in individual 1-day-old adults. At least 10 animals of each genotype were scored. In both oocytes and embryos, when compared to control, the difference between crimson and navy-blue dots is further augmented in *seip-1(A185P)*.

Figure S3. Localization of SEIP-1 in the germline.

(A) Visualization of transgenic wild-type SEIP-1::GFP (*hjSi189*) in 1-day-old *seip-1(-)* adults. GFP is pseudocolored cyan. Dotted lines mark the boundary between different tissues or embryos. Boxed regions were magnified 3x and displayed in the inset. A projection of

4.5 μm z stack reconstituted from 10 focal planes is shown. For anatomical positions of the ROI, refer to Figure 2A.

(B) As in (A), but with transgenic SEIP-1(A185P)::GFP (*hjSi541*).

(C) As in (A), but with transgenic human seipin::GFP (*hjSi223*).

(D) The total number of live progenies from individual animals. At least 10 animals of each genotype were scored. Groups with different letters are significantly different (ordinary one-way ANOVA with Turkey's multiple comparisons test, $p<0.01$). Transgenic SEIP-1::tagRFP is expressed from the single-copy transgene *hjSi434*.

(E) As in (D), but with the percentage of BODIPY-stained embryos quantified in a defined time window. Five independent repeats were scored, each with progeny from four 1-day-old adults.

(F) Schematic representation of endogenously tagged *sec-16A.1 (hj256)*. All isoforms of SEC-16A.1 are fused with C-terminal GFP in *hj256*.

(G) Visualization of SEIP-1::tagRFP (*hjSi434*) and SEC-16A.1::GFP (*hj256*) in a 1-day-old *seip-1(-)* adult. tagRFP and GFP are pseudocolored magenta and cyan, respectively. Single focal planes are shown. Dotted lines mark the boundary between different tissues or embryos. Boxed regions were magnified 5x and displayed at the bottom.

(H) As in (G), but with an animal that expressed SEIP-1::tagRFP and VIT-2::GFP (*crg9070*).

Figure S4. Somatic fatty acyl-CoA desaturases contribute to eggshell integrity.

(A) The biosynthetic pathway of polyunsaturated fatty acids (PUFAs) in *C. elegans*.

(B) The percentage of BODIPY-stained embryos in a defined time window upon constitutive depletion of each gene. Five independent replicates were performed, each with progeny from four 1-day-old *seip-1(-)* adults. Statistical significance on top of each bar is calculated by

comparing with the control group (one-way ANOVA with Dunnett's multiple comparisons test). ns, not significant; ****$p < 0.0001$.

(C) As in (B), but with individual genes knocked down in the specified tissues of one-day-old wild-type adults. Statistical significance on top of each bar was calculated by comparing each experimental group with its counterpart in the control group (RNAi vector) (two-way ANOVA and Sidak's multiple comparisons test).

(D) As in (C), but with *seip-1(-)* adults.

(E) As in (D), but with *plin-1(-); seip-1(-)* adults.

Figure S5. The mRuby::PLIN-1 fusion protein is not fully functional.

(A) Schematic representation of the modified *plin-1* locus by CRISPR/Cas9. All isoforms of PLIN-1 are fused with mRuby at the N-terminus in animals that carry the *plin-1(hj249)* allele.

(B) The total number of live progenies from individual animals. At least 10 animals of each genotype were scored. Groups with different letters are significantly different (ordinary one-way ANOVA with Turkey's multiple comparisons test, $p<0.01$). Germline rescue of *plin-1(-)* was performed by expressing PLIN-1::GFP from a single-copy transgene, driven by the *sun-1* promoter (*hjSi552*).

(C) As in (B), but with the percentage of BODIPY-stained embryos quantified in a defined time window. Five independent repeats were scored, each with progeny from four 1-day-old adults. Data of the control groups in (B-C) were reproduced from Fig. 1A-B as the measurement was all performed at the same time.

Figure S6. FAT-3 deficiency disrupts SEIP-1 localization and yolk protein transport.

(A) Visualization of endogenous SEIP-1::GFP (*hj140*) in wild-type oocytes. The GFP signal is pseudocolored cyan. Dotted lines mark the boundary between different tissues or oocytes.

Boxed regions were magnified 3x and displayed in the inset. A projection of 4.5 µm z stack reconstituted from 10 focal planes is shown.

(B) As in (A), but in *fat-3(ok1126)* mutant background.

(C) Visualization of VIT-2::GFP (*crg9070*) and the germline-specific plasma membrane marker mCherry::PH(PLC1∂1) (*itIs44*) in a wild-type one-day-old adult with control RNAi knockdown. The image was stitched from 10 single focal planes of different xy positions. The boxed region was magnified 3x and shown on the side.

(D) As in (C), but with a worm subjected to ubiquitous *seip-1* knockdown.

(C) As in (A), but with a worm subjected to ubiquitous *fat-3* knockdown. VIT-2::GFP in the pseudocoelom is indicated by a white arrow.

Figure S7. Minor contribution of lipolysis to the development of *seip-1(-); plin-1(-)* embryos.

(A) Schematic representation of knock-in or mutant alleles used in Figure 4 and S7.

(B) The percentage of BODIPY-stained embryos quantified in a defined time window. Five independent repeats of each genotype were scored, each stemming from four 1-day-old adults. Groups that do not share the same letters are significantly different (ordinary one-way ANOVA with Turkey's multiple comparisons test, $p<0.01$).

(C) A schematic summary of the first two steps of lipolysis.

(D) The total number of live progenies from individual animals. At least 10 animals of each genotype were scored. Groups that do not share the same letters are significantly different (ordinary one-way ANOVA with Turkey's multiple comparisons test, $p<0.01$).

(E) As in (D), but with the percentage of BODIPY-stained embryos quantified in a defined time window. Five independent biological samples were scored, each stemming from four 1-

day-old adults. Data of the control groups in (D-E) were reproduced from Fig. 1A-B as the measurement was all performed at the same time.

(F) Visualization of GFP::ATGL-1 expressed from its endogenous locus (*hj345*) in an otherwise wild-type 1-day-old adult. Dotted lines mark the boundary between different tissues or embryos. Boxed regions were magnified 5x and shown in the inset. GFP is pseudocolored cyan. A projection of 4.5 μm z stack reconstituted from 10 focal planes is shown. Scale bar = 10μm.

(G) As in (F), but in *plin-1(-)* mutant background.

(H) As in (F), but in *seip-1(-)* mutant background.

(I) As in (F), but in *plin-1(-); seip-1(-)* mutant background.

(J) Visualization of GFP::ATGL-1 (*hj345*) and LDP-1::mRuby (*hj289*) in an isolated one-cell stage embryo from a 1-day-old *plin-1(-); seip-1(-)* adult. Dotted lines mark the boundary of the embryo. LDP-1::mRuby serves as a LD marker. GFP and mRuby are pseudocolored cyan and magenta, respectively. A single focal plane is shown. The boxed region was magnified 4x and shown at the bottom. Scale bar = 10μm.

Video S1. Time-lapse monitoring of the perivitelline space (PVS) marker mCherry::CPG-2 (magenta, *hj340*) with the plasma membrane (PM) marker GFP::PH(PLC1∂1) (cyan, *itIs38*) in newly fertilized wild-type embryos. Images were acquired *in utero* at 5-sec intervals. Display rate = 10 frames/second.

Video S2. As in Video 1, but in *seip-1(tm4221)* mutant background.

Video S3. Time-lapse monitoring of SEIP-1::tagRFP (magenta, *hjSi434*) with the plasma membrane (PM) marker GFP::PH(PLC1∂1) (cyan, *itIs38*) in newly fertilized wild-type embryos. The fluorescence signals were merged with bright-field (BF) signals that showed

the position of embryos in the uterus. All images were acquired at 5-sec intervals. Display rate = 10 frames/second.
